# Asiaticoside A for the modulation of 1-TbAd- a potential target and ligand for extensive drug resistance *Mycobacterium tuberculosis*

**DOI:** 10.1186/s13568-023-01616-w

**Published:** 2023-10-13

**Authors:** Komal Tilwani, Abhishek Patel, Mainavi Patel, Pankaj Sojitra, Gayatri Dave

**Affiliations:** 1 P D Patel Institute of Applied Sciences, Charotar University of Science and Technology, Changa, 388421 India; 2 QxP Pharma project and GMP services Private Ltd, Ahmedabad, India

**Keywords:** Extensively drug-resistant, *Mycobacterium tuberculosis*, Asiaticoside A, mRNA expression, Molecular dynamic simulation

## Abstract

In nature, terpene nucleosides are relatively rare, with 1-tuberculosinyladenosine (1-TbAd) being an exclusive feature of *Mycobacterium tuberculosis* (Mtb). The convergence of nucleosides and terpene pathways in the Mtb complex appears to have emerged late in its evolutionary history. 1-TbAd (PDB ID: 3WQK) is a prominent chemical marker for Mtb and may contribute to its virulence-related properties when exported extracellularly. We gathered a comprehensive set of 270 phytochemicals from diverse Ayurvedic texts and treatment traditions. Subsequently, we conducted structure-based molecular docking analyses to identify compounds exhibiting the strongest binding affinity for 1-TbAd, highlighting their potential as drug candidates. These selected compounds were further subjected to an in-vitro growth inhibition assay against the reference strain *Mycobacterium tuberculosis h37rv*. Among the candidates, Asiaticoside A (ASA) emerged as a promising candidate from the pool of 270 compounds. To assess the impact of ASA on 1-TbAd expression, we employed a PCR-based mRNA expression assay, revealing ASA's ability to downregulate 1-TbAd expression in extensively drug-resistant MTb strains. Remarkably, the conventional drug rifampin showed no such effectiveness in our experiments. We further conducted molecular dynamic simulations to explore the interaction between ASA and 1-TbAd in a cellular-like environment, confirming the stability of their interaction. Also, we predicted ASA's stability toward causing inducing the random mutations in the target gene. With this, we propose a novel target and its modulator to treat extensively drug-resistant MTB.

**Graphical Abstract:**

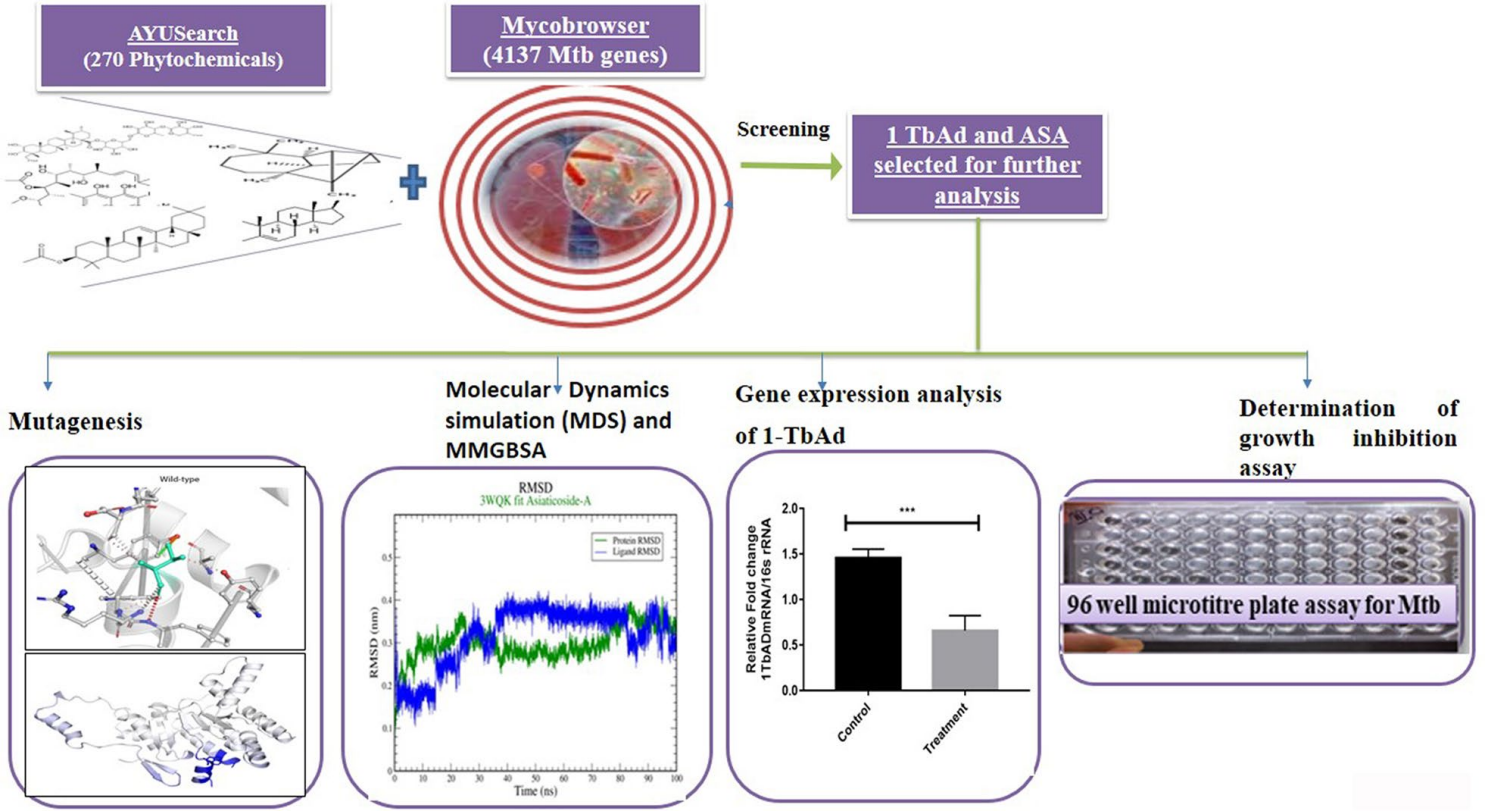

**Supplementary Information:**

The online version contains supplementary material available at 10.1186/s13568-023-01616-w.

## Key points

- In-silico screening studies for identifying the hit compound
- mRNA expression analysis for validation of hit compound
- Random in-silico mutagenesis and Molecular dynamic simulation studies for evaluating the efficiency of Asiaticoside A.

**Supplementary Information:**

The online version contains supplementary material available at 10.1186/s13568-023-01616-w.

## Introduction

Despite various nationwide tuberculosis (TB) eradication programs in the twenty-first century, tuberculosis is still a leading cause of mortality and morbidity worldwide. Tuberculosis (TB) is one of the top 13 causes of death worldwide and the most common infectious disease (Gong et al. 2020).TB can be cured if therapy is started immediately, followed correctly, and continued throughout the 6–9 month treatment period. Unfortunately, inconsistent treatment, intermittent stoppage, non-adherence to medication, and lack of disease information collectively contribute to failure in TB eradication (Skripconoka et al. 2013). In India, the standard short-course therapy for all categories of drug-sensitive tuberculosis (DS-TB) comprised a 6-month regimen, with eight weeks of the intensive phase of the drugs Isoniazid (H), Rifampicin (R), Pyrazinamide (Z) and Ethambutol (E) (Chaw et al. 2020) and a sixteen-week continuation phase of three medications Isoniazid, Rifampicin and Ethambutol. The root cause is rapidly evolving drug resistance in tubercle bacilli, multidrug resistance (MDR-TB) to extensively drug-resistant (XDR), and sometimes exhibits total drug resistance (TDR). These drug-resistant variants of the bacterium pose a severe threat to humankind. So far, TB has been effectively managed in developed countries. Conversely, the developing countries still have difficulty controlling the spread since drug-resistant variants are crippling their efforts.

In *Mycobacterium tuberculosis* (Mtb), drug resistance can be explained by two main factors and their interactions: extrinsic factors related to the social determinants of TB in populations and the quality of TB control and prevention services and intrinsic factors resulting from the acquisition of mutations in drug-resistant genes (Allué-Guardia et al.^2021^). Unlike other bacterial pathogens, harbouring drug resistance through horizontal gene transfer is not consistently reported in Mtb. However, evidence suggests that, at least for some anti-TB drugs, acquired drug resistance results from a stepwise acquisition and fixation of mutations leading to a gradual increase in resistance, initiated with the addition of isoniazid resistance, subsequently followed by rifampicin or ethambutol resistance (Beraldi-Magalhaes et al. 2021).

In addition to resistance caused by target mutations, several specific mechanisms of anti-Mtb innate resistance have also been described. These include limited drug accessibility to the target due to the low permeability of the Mtb cell envelope, modification of drugs by Mtb enzymes, and the existence of Mtb efflux pumps removing drugs that can cross the Mtb cell envelope. As a result of MDR and XDR, the conventional six- to nine-month treatment has become less active, time-consuming, and costly(Arora et al.^2021^). To prevent the reactivation of the infection, new antituberculosis medications must be developed that can inhibit both actively reproducing bacilli and a non-growing permanent population of MTB (Mishra et al. 2021).

We first reviewed Mtb gene targets and their possible modulators (Table 1). Next, we scrutinized the Mtb gene targets and ligands from various organism-specific databases. Then, we used molecular docking and subsequent binding interaction analysis to weed out the weak interaction. The point mutation is one of the main factors in elevating drug- resistance. Hence, we performed a mutational analysis to identify the key residues.

**Table 1:** Ligand-target binding affinity score

No	PDB	Ligand	Binding affinity(kcal/mol)
1	1MOP	Madasiatic acid	− 11
2	3WQK	Asiaticoside A	− 11.1
3	6VPT	Asiaticoside A	− 9.9
4	1Y8T	Longicyclene	− 10
5	3AJA	Longicyclene	− 9.9
6	6OR2	Asiaticoside A	− 9
7	5OEQ	Madasiatic acid	− 10.1
8	2KHR	β-sitosterol	− 10.9
9	2VFW	Cystargamide B	− 10.9
10	2VG2	Myristic acid	− 10
11	1V0J	Palmitic acid	− 9.8
12	7AHB	Kalmeghin	− 9.6
13	1TPY	Oleanolic acid	− 10.2
14	3QJ7	Oleanane	− 10.5
15	1U5V	Amyrin	− 9.3

Further, the molecular dynamic simulation was used to investigate ligand-target stability at various physiological conditions. As a result, Asiaticoside A (ASA) was identified as a potential growth inhibitor for XDR Mtb. Asiaticoside A is commonly found in *Centella Asiatica*. *Centella Asiatica*, also known as Gotu Kola, is a medicinal herb widely recognized for its pharmacological properties, including anti-inflammatory, antioxidant, and antimicrobial activities.

## Methodology

### Microbial samples and other materials

Mtb H37Rv was initially isolated from a patient with pulmonary tuberculosis in 1905 by Albert Calmette and Camille Guérin at the Pasteur Institute in Paris, France. The strain was maintained and distributed by American Type Culture Collection (ATCC 27294) centre. For our study, a clinical sample from extensively drug-resistant *Mycobacterium tuberculosis h37rv* was obtained from the district Tuberculosis laboratory (Guna, Gujarat, India). It has a rpoB gene mutation (gene accession number: JN037845) and has resisted the first, second, and third lines of TB drugs in primary screening. In addition, we also obtained a sample for drug-sensitive tuberculosis from the same laboratory. The culture was then inoculated into the Middlebrook medium (Himedia) supplied with 0.05% Tween 80 and albumin-dextrose-catalase. The culture was maintained by transferring in the fresh medium every 30 days. Madasiatic acid, Asiaticoside A(ASA), β-sitosterol, Cystargamide B, Myristic acid, Palmitic acid, Kalmeghin, Oleanolic acid, Longicyclene, Oleanane, and Amyrin were purchased from Sigma-Aldrich.

### Screening of gene targets and ligands specific to Mtb

Mycobrowser is the repository for genomics and proteomic data obtained from pathogenic mycobacteria, displaying 4173 genes for the chosen strain. *Mycobacterium tuberculosis* (Mtb) establishes human infection through various mechanisms, mainly involving the three sets of gene products. First, we grouped the genes based on a cellular role, such as multiplication, cell-wall biosynthesis, and mycolic lipid synthesis. After that, we manually investigated each gene product for its experimentally validated ligand. Then, we identified the fifteen unmapped targets (ligands are not validated). First, the protein structures were obtained from the protein data bank (PDB) (Additional file 1: Table S2) and processed to remove the bound cofactors, water molecules, ions, and nonspecific molecules using the UCSF Chimera version 1.14 (Pettersen et al.^2004^). Finally, the protein structure was saved in PDB format for further analysis.

Next, we investigated the various databases of Traditional Indian medicinal plants, such as IMPPAT, AYUSearch (Tilwani et al. 2023) and Ayurvedic pharmacopoeia. The queried keywords were tuberculosis (kshay rog) and antibacterial activity. We identified 270 phytochemicals, and their chemical structures were obtained from PubChem (Kim et al.^2019^) (Additional file 1: Table S3). We used the 3D structure for selected chemotypes for input in Version 1.14 of UCSF Chimera. The model was built using Chimera's structure builder option from their respective CID (Huang et al.^2015^). The 3D structures were checked for the number of hydrogens and saved as separate.pdb files.

### In-silico and in-vitro screening of compounds with antimycobacterial activity

The library for 270 Chemotypes was then used for docking with 15 selected gene targets. For docking, we used AutoDock 4.22 (Trott and Olson 2010). We performed 4,050 individual docking studies for each permutation and identified the crucial interactions. The protein–ligand complex was visualized in PyMOL (Yuan et al 2017). Binding sites for each target were predicted using AutoGridFR (Zhang et al.^2019^), a platform for predicting docking sites on protein receptors. Also, the crucial amino acids involved in ligand interaction cited in the primary citation of the receptor and other similar reports were taken into account in the docking site selection. Grid coordinates are generated and used for molecular docking.

The in-vitro antimicrobial assays using the well-diffusion method were performed for each (stock 1 mg/ml) of the ten phytochemicals; only the ASA gave a notable zone of inhibition at the studied concentration and hence opted for further optimization. The MIC of ASA was determined by inoculating the 50 µl (2×10^6^ cells/ml) of activated XDR Mtb culture in 100 µl of cultivation media. Initially, the gradient of ASA, 1-5 µg of concentration, was created in a microtiter plate. Each set was performed in triplicates, and the ASA-free well was considered a negative control. The covered microtiter plate was incubated at 37 ºC for 24 h. The absorbance was recorded at 600 nm in the plate reader (Biotek, USA). The Kanamycin (MIC: 0.78 µg/ml) and Rifampin (MIC: 1 mg/ml) were tested in separate wells as a positive control for the study.

### Quantitative gene expression analysis

The effects of ASA on 1-TbAd expression was surveyed by introducing five micrograms of ASA into a cultivation medium containing a high-density culture of extensively drug-resistant (XDR) Mtb (2*10^6^ cells/ml). After a 24-h incubation period, total RNA was extracted from the treated cultures using TRIzol reagent (Invitrogen, Carlsbad, CA, USA), and the purity of the RNA template was assessed using a NanoDrop ND-1000 spectrophotometer (Thermo Fisher Scientific, Wilmington, DE, USA). Subsequently, we designed gene-specific primers for our target gene (Additional file 1: Table S1), 1-TbAd. We estimated the template concentration using reverse transcription quantitative PCR (RT-qPCR) on a LightCycler 480 II Real-Time PCR System (Roche Diagnostics GmBH) with the 2X OneStep qRT-PCR Mastermix Kit (PrimerDesign Ltd., Southampton, UK), following the manufacturer's instructions. The RT-qPCR protocol included reverse transcription at 55 °C for 10 min, enzyme activation at 95 °C for 2 min, followed by 50 cycles of denaturation at 95 °C for 10 s and annealing at 60 °C for 60 s.

### Molecular dynamics simulation (MDS) and MMGBSA

The GROMACS package program (version 2022.2) was used to simulate the stability of a docked complex in a natural cell environment (Ganesan et al. 2017). Ligand topology was created using the SwissParam server (Aalten et al. 1996), and protein topology was created using pdb2gmx and the CHARMM27 force field (Schmid et al 2011).

The complexes were inserted into the system after the force field was applied. They were solvated in a cubic box greater than 1 nm from the protein's edge using the SPC water model (Fawzi et al. 2022) with periodic boundary conditions. The system was neutralized by adding Na + ions, and the steepest descent algorithm was used to minimize energy for 50,000 steps. The system was then equilibrated using 100 ps of NVT simulation at 300 K and 100 ps of NPT simulation. In the constant-temperature, constant-pressure (NPT) ensemble, the Leapfrog algorithm was used to separately couple each component such as protein, ligand, water molecules, and ions (Van Gunsteren and Berendsen 1988). To keep the system stable (300 K temperature and 1 bar pressure), the Berendsen temperature and pressure coupling constants were set to 1 and 2, respectively (Berendsen et al. 1995). Finally, a 100-ns MD simulation was performed in an isothermal and isobaric conditions ensemble at 300 K. To keep the pressure constant at 1 bar, the pressure coupling with time constant was set at 1 ps, and the LINCS algorithm (Hess et al. 1997) was used to constrain the bond lengths. At 1.2 nm, the Van der Waals and Coulomb interactions were truncated; the PME algorithm built into GROMACS was used to reduce the error from truncation.VMD (Visual Molecular Dynamics) 1.9.2 (Humphrey et al. 1996) is used to visualize the trajectory files, and HeroMDAnalysis (Rawat et al. 2021).

Using the Prime module and the Molecular Mechanics software: Generalized Born model and Solvent Accessibility (MM-GBSA), the binding free energies for all complexes were calculated. Total G binding was calculated by examining each protein–ligand complex's free solvation energy (polar and nonpolar solvation energies) and potential energy (electrostatic and Van der Waals interactions) (He et al. 2020). During the binding process, the Prime MM-GBSA computed bound and unbound molecules using the VSGB2.0 implicit solvent model and the OPLS2005 force field.

### Mutagenesis and stability study

The mutation alters the protein structure, where the position of mutation determines a protein's active or inactive state. Mutation at the active binding pocket remarkably impacts protein binding and functions. Hence, our study identified the active site for our target proteins and residues at active sites. Subsequently, we randomly altered one or two nucleotides of crucial residues (Additional file 1: TableS S5 and S6). Also, we have considered the MD simulation results to choose the residues for mutation. Finally, the DynaMut webserver (Rodrigues et al. 2018) was used to test the stability of the mutant protein structure. Next, the selected phytochemicals were re-docked with the altered protein structures to better understand the residue's role in the binding pocket.

## Results

### Ligand screening; in-silico and MIC assays

The lower Ligand-target binding affinities are considered, the better fit. Therefore, an interaction with a binding affinity score of − 9 or above was selected for further MIC studies **(**Table 1**)**. Of 10 such compounds, ASA gave higher binding affinity towards the multiple Mtb-specific targets: 1MOP, 3WQK, and 6OR2. The gene product of 1MOP catalyzes the condensation of pantoate with beta-alanine, which is essential for synthesizing vitamin B_5._ Ciulli et al. (2015) reported it as a potential antimicrobial target. The target protein 6OR2 synthesizes the Mtb membrane (Bolla 2020). The next target, 1-tuberculosinyladenosine synthase (3WQK), is lysosomotropic and causes gross phagosome disruption in the host through that escape from digestion by lysosomal enzymes. Therefore, all the above targets can be potential protein hits for the development of drugs. However, among them, 3WQK is relatively novel as a drug target, and no inhibitor has been reported. We pulled it for further gene expression studies.

Next, we performed the growth inhibition assay on XDR Mtb. ASA gave almost 90% growth inhibition, while no other phytochemicals gave a significant bacteriostatic effect. The earlier reports have established ASA as a neuroprotective agent that can cross the blood–brain barrier (Hanapi et al. 2021). Further, Michael et al. (2021) have shown the immunomodulatory activity of macrophages infected with *S. flexneri*, and have also noted the improvement in the killing action of macrophages. In our findings, the ASA is a potent antibacterial compound that gave 90% growth inhibition **(**Table 2**)** on extensively drug-resistant tubercle bacilli and 100% on reference strain *E. coli*. Here, we further screened ASA for gene expression analysis.

**Table 2:** MIC and percentage of growth inhibition (only the significant results are represented here)

Agent	MIC µg/ml	Percentage Inhibition
ASA	5	+ + + +
Kenamycine	0.78	+
Rifampin	1000	+ +

The growth inhibition was calculated using the following formula: Growth Inhibition (%) = $\frac{\mathrm{Control}-\mathrm{Test}}{\mathrm{Control}}\times 100$

### Gene expression analysis and Target validation

**Fig. 1:**
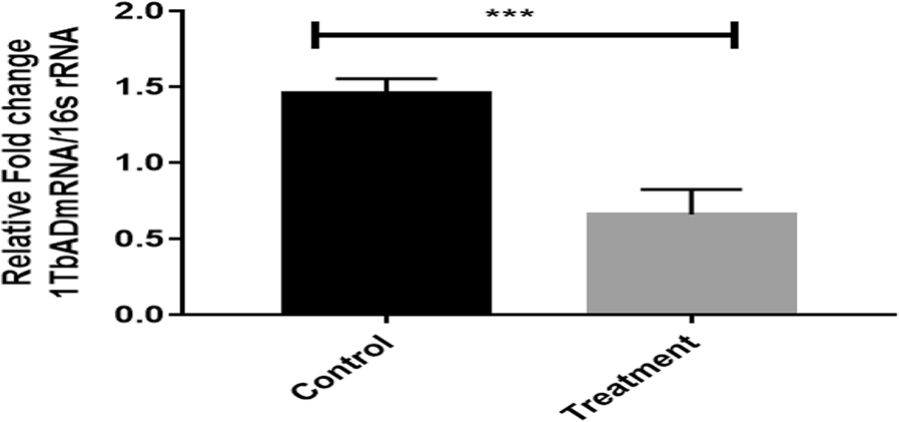
The fold change of gene expression (relative to control) with a significant difference at ***P < 0.001

The RT-qPCR assay was used to investigate the relative fold change in the expression of 1-TbAd post-ASA treatment. The fold change was computed using C_t_ values for treated and un-treated XDR suspension using the 2^−∆∆CT^ method. The ASA-treated sample showed a relative one-fold change compared to the untreated control (Fig. 1**)**. An apparent assumption indicates the ASA-mediated down-regulation of the 1-TbAd could be one of the mechanisms for the ASA-induced growth inhibition.

### Molecular dynamics simulation

**Fig. 2:**
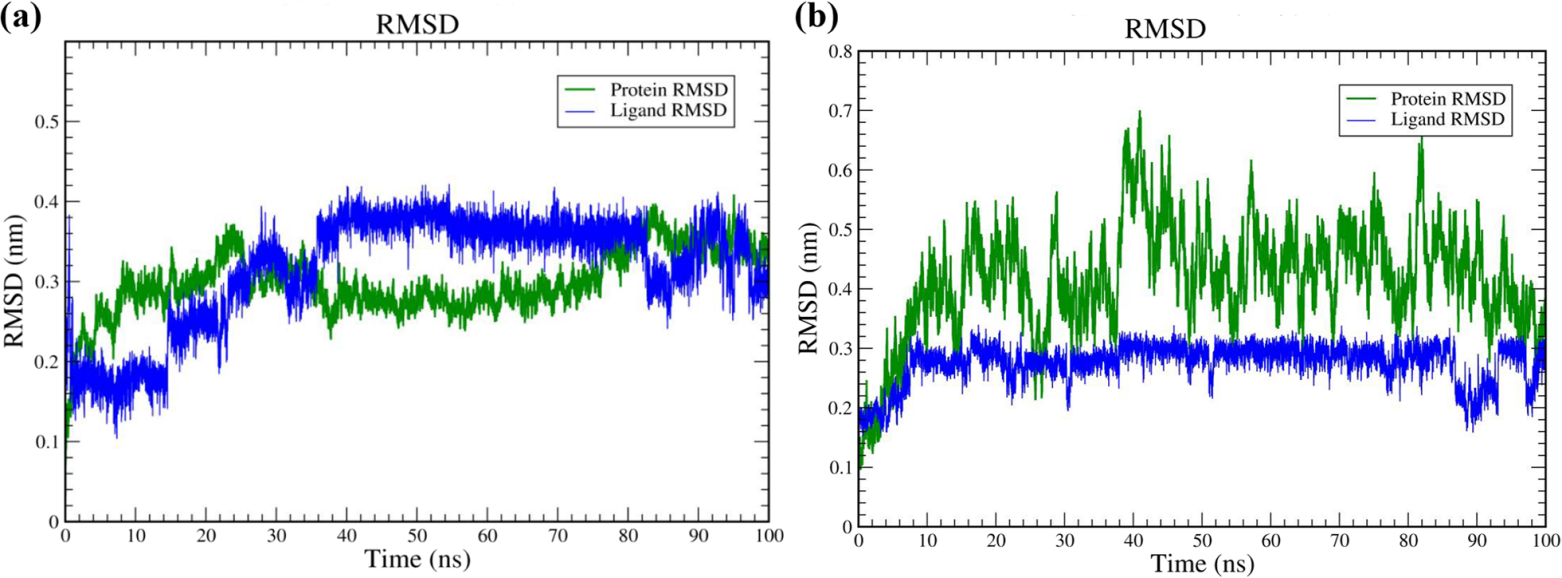
Lig Fit Plot RMSD graph **a** 3WQK_ASA **b** rpoB_Rifampin

The best-docked complex of the molecular docking study was simulated to predict the in-vivo stability of the ligand and target. The complex of the ASA-3WQK had a binding score of -11.8 kcal/mol, and the direct contact of multiple amino acid residues was observed. If the simulation has stabilized, RMSD analysis can show that the changes at the end of the simulation are centred on a thermally and energetically stable conformation. From the obtained trajectory analysis, the RMSD of protein Carbon atom (Ca), backbone, and heavy atoms were observed in the range of 0.1–0.3 nm and 0.1–0.7 nm, respectively. The adjacent Cα atoms fluctuated in the range of 10 to 25 ns and finally equilibrated after 30 ns of simulation with an average RMSD value of 0.3 nm. However, as the protein's size increases, this value range widens. ASA's average LigFitLig RMSD is less, indicating that the molecules did not undergo significant rotation. A lock-and-key type of phenomenon involving ligand binding in the protein cavity, Ligfit Prot values are easier to interpret. The LigFit Prot graph of our hit (Fig. 2a**)** and reference standard (Fig. 2b**)** can be seen in Fig. 2, where ligand RMSD changes based on protein backbone translation. Figure 2 clearly illustrates that the calculated RMSD of the protein backbone is aligned to Ligand RMSD, indicating the ligand's stability to the protein and binding pocket.

The 3WQk-ASA RMSD plots illustrate structural fluctuations around 0.3 nm. In many cases, a system's structural stability (robustness) is not required for its thermodynamic stability (binding affinity) (Du et al 2016). Here, the 3WQk-ASA pair has higher binding affinities (− 11.1) than the rpoB-Rifampin.

**Fig. 3:**
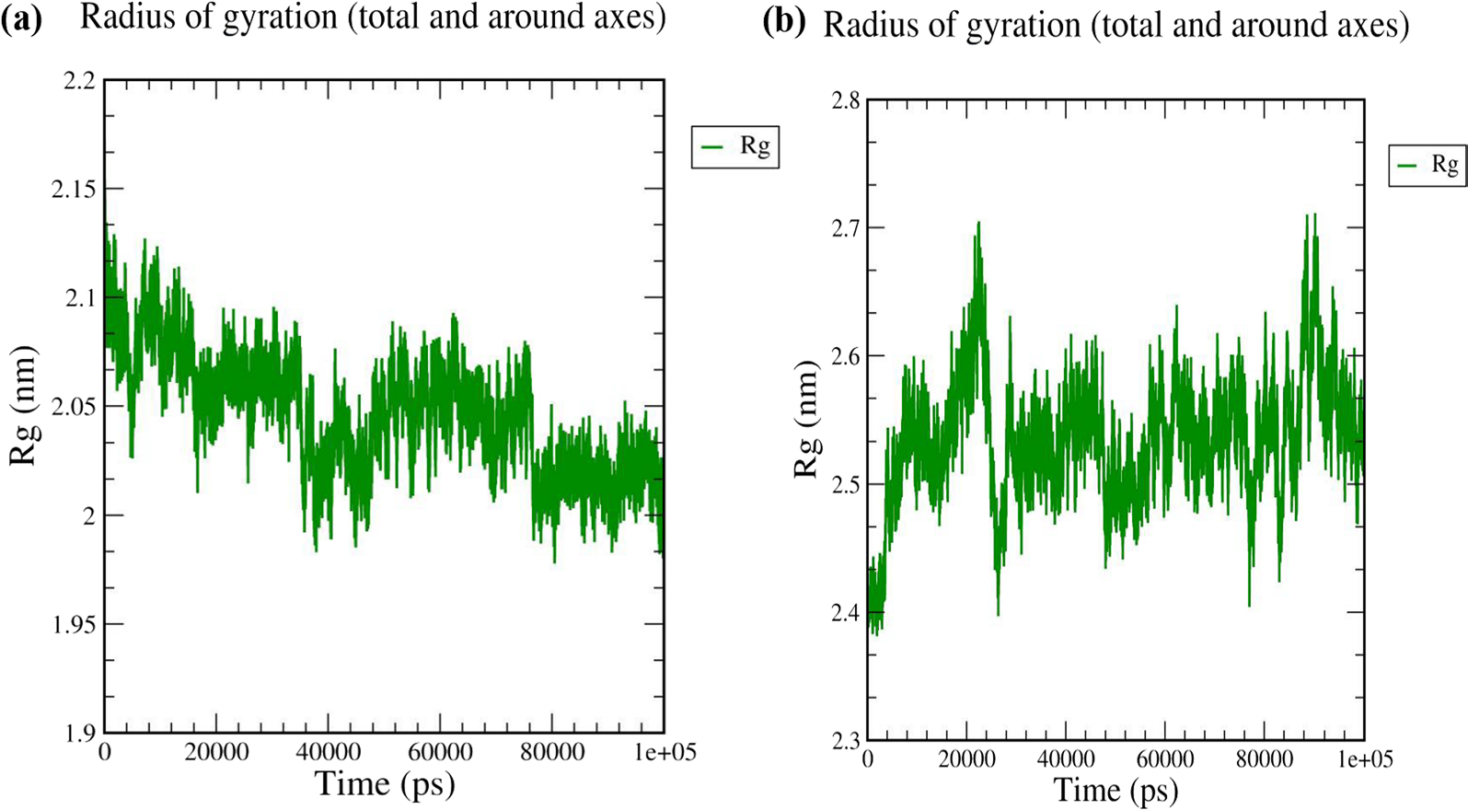
A plot for Radius of Gyration **a** 3WQK_ASA **b** rpoB_Rifampin

The radius of gyration (Rg) allows us to assess the changes in compactness of a ligand–protein complex. Finally, higher Rg reduces the compactness of the ligand–protein complex. During the MD simulation, Rg is used to determine if the complexes are stable, folded or unfolded. 3WQK-ASA has an average Rg value of around 2.05 nm (Fig. 3a). Furthermore, the Rg value of the Rifampin-rpoB complex was 2.4 nm, which is very similar to the reference molecule. (Fig. 3b).

The solvent-accessible surface area measures the interaction between complexes and solvents (SASA). For ligand–protein complexes, SASA was calculated to determine the extent of conformational changes that occurred during the interaction. The SASA value vs. time plot for each protein–ligand combination is shown in Additional file 1: Fig. S1a. The average SASA for complex 3WQk-ASA was 180 nm2, whereas the average SASA for complex rpoB-Rifampin was 210 nm2 (Additional file 1: Fig. S1b). These calculations show that the 3WQk-ASA has a very similar value to SASA as the reference complex. We discovered that the protein-ligands complex's SASA values for the 100 ns MD simulation are largely constant, indicating that the protein structure has not changed significantly.

### MMGBSA analysis

The calculation of binding free energy change provides information about the ligand's ability to interact strongly with the amino acids of the protein. The energy released (∆G binding energy) as a result of bond formation, or rather ligand–protein interaction, is in the form of binding energy and determines the stability of any given protein–ligand complex. A favorable reaction has a negative free energy. Table 3 shows the contributions to total energy from various components such as hydrogen-bonding correction, lipophilic energy, pi-pi packing correction, and van der Waals energy in addition to total energy.

**Table 3:** Binding free energy calculation using MMGBSA approach

Compound	MMGBSA-∆G-binding energy (Kcal/mol)	MMGBSA-∆G-Coulomb energy (Kcal/mol)	MMGBSA-∆GHbond (Kcal/mol) (Kcal/mol)	MMGBSA-∆G Lipophillic energy (Kcal/mol)	∆GPacking (Kcal/mol) (Kcal/mol)	∆Gvander waal energy (Kcal/mol) (Kcal/mol)
ASA	− 90.07	− 33.45	− 3.45	− 65.52	− 5.37	− 66.47
Rifampin	− 82.88	− 54.33	− 4.07	− 59.78	− 3.88	− 52.38

It is discovered that ASA have a negative ∆GBinding energy. The lead molecule ASA had an MM-GBSA score of -90.07, whereas the reference molecule had a significantly lower MM-GBSA score of -80.82. Based on these findings, it is possible to conclude that ASA had a higher free binding energy score than the reference compound. The compound has similar energies corresponding to van der Waals interactions, indicating that it prefers to be near the interacting amino amides of 3WQK. The compound has negative Coulomb energy values, which describe potential energy in systems.

### Prediction of mutation hot spot for ASA-3WQK

Though many drug candidates have been tested for MTb and showed considerably good antibacterial activities, in most of the drugs, drug-resistance phenomena were noted later (Maddila et al 2020). As discussed in the introduction section, the primary reason for evolved drug resistance in Mtb is a point mutation obliterating the drug effectiveness. Therefore, we computed the ligand-target interaction stability by inducing the target's active binding pocket mutation. First, the signature residues interacting with ASA were chosen from MD simulation studies. Second, using the Autogrid binding site prediction suit, we predicted five possible ASA and Rifampin binding pockets for 1-TbAd and rpoB, respectively **(**Table **S7).** Next, we identified the common residues that likely play a role in direct association with ASA and Rifampin. For ASA-1-TbAd that are Gly35, Thr36, and Tyr90, we replaced them with the residues of the same group (polar to polar), e.g. Glycin35 with the Cysteine gave double change in Gibbs free energy (ΔΔG) at 0.817 kcal/mol representing the stable interactions shown in Additional file 1: Table S5. Another few changes were found unfavorable when Threonine (Thr) replaced Tryptophan (Trp); the change in vibrational entropy energy (ΔΔSVib ENCoM) was -1.326 kcal/mol^−1^K^−1^. It showed that Thr to Trp change in 1-TbAd resulted in rigid structure and reduced molecular flexibility (Additional file 1: Fig. S2). In summary, out of 15 changes, we found eight destabilizing interactions mainly associated with the replacement of Tyr90 shown in Additional file 1: Table S5. For Rifampin-rpoB interaction, Arg222 is a common and crucial residue, and any point mutation here will result in destabilized ligand-target interaction shown in Additional file 1:Table S6.

Additionally, the ligand-binding affinities to mutated versions were evaluated by docking studies. The result shows some non-significant increase in binding affinity in a few sets for the scrutinized association. However, overall, the ligand-target interaction is robust and stable towards the residue changes for ASA.

## Discussion

Butler et al. (2019) tested patient samples and recorded the presence of 1-TbAd in almost all Mtb-infected patients in notable concentrations. Also, they have proposed 1-TbAd as a possible marker for the diagnosis of TB. Here, we elucidated 1-TbAd as a drug target and proposed a hypothesis that suppression of 1-TbAd may result in growth inhibitions. However, the suppression of 1-TbAd may not only be sufficient for achieving complete growth inhibition. Hence, 1-TbAd inhibitors can be a candidate for combinatorial drug therapy, whereas, with other drugs, the presented inhibitor help achieve the growth restrictions for antibiotic-resistance strain. We also tested the Rimfapin and Kanamycin for the possible suppression of 1-TbAd in XDR-Mtb, but no changes in the gene transcription have been noted.

*Centella Asiatica* is a traditional medicinal plant famous in Asian folklore. ASA was extracted from the plant and speculated to promote wound healing (Lee et al 2012) by accelerating the cell migration. Our finding shows that ASA interacts with membrane lipid transporter (6OR2), diterpene cyclase (6VPT), and 1-TbAd, where it down-regulates the 1-TbAd. It would be interesting to investigate the impact of ASA on the other two targets in the future. The multiple-target interaction is more beneficial for a drug candidate than a ligand with a single target because a point mutation on the target often renders the therapeutic potential. Earlier, ASA was reported to modulate the expression of IL-1β in macrophage studies for rat models (Kimura et al. 2008). The cell permeability and localization of ASA in macrophages are well established. Therefore, ASA could be a promising alternative for recognized antibiotics that failed to inhibit our chosen microbial strain. Also, the in-silico mutagenesis study established the 1-TbAd as a putative target for the ASA, except Tyr90, the *interaction* is fairly stable with many other point mutations. With this, the in-silico and in-vitro assays have recognized the ASA as a putative lead compound for the 1-TbAd. The target's robustness can be evaluated in the future by performing a site-directed mutagenesis study. The research presents an alternate opportunity for combating the drug-resistant XDR Mtb, otherwise untreated.

In conclusion, we first reviewed Mtb gene targets and their possible modulators (Table 1). Then, we used molecular docking and subsequent binding interaction analysis to weed out the weak interaction. The point mutation is one of the main factors in elevating drug- resistance. Hence, we performed a mutational analysis to identify the key residues. Further, the molecular dynamic simulation was used to investigate the stability of complex. As a result, Asiaticoside A (ASA) was identified as a potential growth inhibitor for XDR Mtb. Also, through the results of the in-silico study, we present tuberculosinyladenosine synthase (1-TbAd) as a potential target of ASA. Next, the gene expression analysis has shown that ASA is downregulating the 1-TbAd.

### Supplementary Information

**Additional file 1: Table S1.** Commercial drugs; targets and their mode of action. **Table S2.** Active chemical constituents and their associated identifier of the traditional herbs. **Table S3.** Mapped gene targets with a significant role in MTB aetiology. **Table S4.** Primers for 1TbAd. **Table S5.** Mutation in the familiar residue of the 1-TbAD. **Table S6.** Mutation in the familiar residue of the rpoB. **Table S7.** Prediction of the active binding pockets for 1-TbAd-3WQK. 14. **Figure S1.** A plot SASA Value **A** 3WQK_ASA **B** rpoB_Rifampin. **Figure S2.** **a** Wild type **b** Color codes are displayed on the right panel, and mutant residue is colored in the light-green and depicted in the stick. **c** The vibrational entropy change that occurs during mutation causes amino acids to change color. The rigidification of the structure is represented by the color blue.

## Data Availability

The dataset used in the study available in public rpository. The other data which we were analyzed through our finding also available as suplementary material.
