# Supplementary material for: Asiaticoside A for the modulation of 1-TbAd- a potential target and ligand for extensive drug resistance Mycobacterium tuberculosis
Source: AMB Express. 2023 Oct 13;13:111. doi: 10.1186/s13568-023-01616-w (PMC10575824; doi:10.1186/s13568-023-01616-w)
Supplement: Supplementary file 1 — Additional file 1: Table S1. Commercial drugs; targets and their mode of action. Table S2. Active chemical constituents and their associated identifier of the traditional herbs. Table S3. Mapped gene targets with a significant role in MTB aetiology. Table S4. Primers for 1TbAd. Table S5. Mutation in the familiar residue of the 1-TbAD. Table S6. Mutation in the familiar residue of the rpoB. Table S7. Prediction of the active binding pockets for 1-TbAd-3WQK. 14. Figure S1. A plot SASA Value A 3WQK_ASA B rpoB_Rifampin. Figure S2. a Wild type b Color codes are displayed on the right panel, and mutant residue is colored in the light-green and depicted in the stick. c The vibrational entropy change that occurs during mutation causes amino acids to change color. The rigidification of the structure is represented by the color blue. [file 13568_2023_1616_MOESM1_ESM.pdf]

Additional file 1Table

Table S1 Commercial drugs; targets and their mode of action

No	Drug	mode of action	Putative mutant targets	Drug usage
1	Rifampicin (RIF)	Inhibits RNA polymerase activity	rpoB,rpoA,rpoC	1st line (Swain et al.2020)
2	Isoniazid (IHN)	Inhibition of cell wall synthesis	kasA, katG, inhA,AhpC,niA	
3	Pyrazinamide (PZA)	Bacteriostatic/Bactericidal	pncA, rpsA	
4	Ethambutol (EMB)	Bacteriostatic	embB, ubiA,embR,embA,	
5	Moxifloxacin,Gatifloxacin	Anti-topoisomerase activity	gyrA, gyrB	2nd line (Cohen et al,2019)
6	Para-amino salicylic acid	Bacteriostatic	thyA,folC,dfrA,ribD	
7	Kanamycin / Amikacin	Bactericidal	rrs, eis, whiB7	
8	Capreomycin (CAP)	Inhibition of protein synthesis	rrs, tlyA	
9	Ethionamide	Bacteriostatic	KasA,inhA,ethA,ethR	
10	Cycloserine	Inhibition of cell wall synthesis	alr,ddl,cycA	

11	Clofazimine	Bacteriostatic	rv0678,rv1979c,ndh,pepQ	3rd line (Swain et al,2020)
12	Bedaquiline	Inhibition of protein synthesis	rv0678,atpE,pepQ	
13	Delamanid	Bactericidal	fbiC,fgd1,fbiB,fbiA	
14	Linezolid	Inhibition of protein synthesis	rplC,rrl	

Reference

1. Cohen KA, Manson AL, Desjardins CA, Abeel T, Earl AM (2019). Deciphering drug resistance in Mycobacterium tuberculosis using whole-genome sequencing: Progress, promise, and challenges. Genome Medicine, 11(1), 1–18.

2. Swain SS, Sharma D, Hussain T, Pati S (2020). Molecular mechanisms of underlying genetic factors and associated mutations for drug resistance in Mycobacterium tuberculosis. Emerging Microbes & Infections, 9(1), 1651–1663.

Table S2 Active chemical constituents and their associated identifier of the traditional herbs

No	Indian traditional herbs	Chemical constituents	PubChem ID
1	*Kaempferia galanga L*	Ethyl p-methoxycinnamate	5281783
2	*Kaempferia galanga L*	4-Methoxycinnamic acid	699414
3	*Kaempferia galanga L*	Ethyl cinnamate	637758
4	*Kaempferia galanga L*	Methyl (2R,3S)-2,3-dihydroxy-3-(4-methoxyphenyl) propanoate	11020577
5	*Kaempferia galanga L*	Ethyl (2R,3S)-2,3-dihydroxy-3-(4-methoxyphenyl) propanoate	10681490
6	*Kaempferia galanga L*	(−)-Sandaracopimaradiene	443469
7	*Kaempferia galanga L*	Kaempulchraol I	73946812
8	*Kaempferia galanga L*	Kaempulchraol E	122397599
9	*Kaempferia galanga L*	Kaempulchraol L	127034336
10	*Kaempferia galanga L*	Kaempferol	5280863
11	*Kaempferia galanga L*	Kaempferide	5281666
12	*Kaempferia galanga L*	Glucuronic acid	94715
13	*Kaempferia galanga L*	Galacturonic acid	439215
14	*Kaempferia galanga L*	Ferulic acid	445858
15	*Kaempferia galanga L*	*trans-p*-Hydroxycinnamic acid	637542

16	*Kaempferia galanga L*	*p*-Hydroxybenzoic acid	135
17	*Kaempferia galanga L*	*p*-Methoxybenzoic acid	7478
18	*Kaempferia galanga L*	Methyl 3,4-dihydroxybenzoate	287064
19	*Kaempferia galanga L*	Vanillic acid	8468
20	*Kaempferia galanga L*	Cystargamide B	139589531
21	*Adhatoda vasica*	Vasicine	72610
23	*Adhatoda vasica*	Deoxyvasicine	442894
24	*Adhatoda vasica*	vasicinolone	158720
25	*Adhatoda vasica*	Betain	4682572
26	*Adhatoda vasica*	7- hydroxyvasicine	4486241
27	*Adhatoda vasica*	Vasicol	92470596
28	*Adhatoda vasica*	Vasicoline	626005
29	*Adhatoda vasica*	Vasicolinone	627712
30	*Adhatoda vasica*	Anisotine	442884
31	*Adhatoda vasica*	Apigenin	5280443
32	*Adhatoda vasica*	Astragalin	5282102
34	*Adhatoda vasica*	Quercetin	5280343
35	*Adhatoda vasica*	Vitexin	5280441
36	*Saussurea lappa*	Costunolide	5281437
37	*Saussurea lappa*	β-costic acid	12304100
38	*Saussurea lappa*	Reynosin	482788
39	*Saussurea lappa*	Syringin	5316860
40	*Saussurea lappa*	Chlorogenic acid	1794427
41	*Saussurea lappa*	Dehydrocostus lactone	73174
42	*Saussurea lappa*	Cynaropicrin	119093
43	*Saussurea lappa*	Soulangianolide A	101320297
44	*Saussurea lappa*	Syringaresinol	100067
45	*Saussurea lappa*	Scopoletin	5280460
46	*Saussurea lappa*	Inulin	24763
47	*Saussurea lappa*	sesquiterpene lactone	314621
49	*Saussurea lappa*	Camphene	6616
50	*Saussurea lappa*	Costol	12304104
51	*Saussurea lappa*	β-sitosterol	222284
52	*Saussurea lappa*	β-stigmasterol	5280794
53	*Saussurea lappa*	Betulin	72326
54	*Saussurea lappa*	Aplotaxene	5352710
55	*Saussurea lappa*	β-elemene	6918391
56	*Pinus longifolia*	Longifolene	289151
57	*Pinus longifolia*	Palmitic acid	985
58	*Pinus longifolia*	Longicamphenylone	91747202
59	*Pinus longifolia*	humulane-1,6-dien-3-ol	5353015
60	*Pinus longifolia*	α-Thujene	17868
61	*Pinus longifolia*	α-Pinene	440968

63	*Pinus longifolia*	1-Isopropyl-4-methylenebicyclo[3.1.0]hex-2-ene	524198
64	*Pinus longifolia*	1,2,4-Trimethylbenzene	7247
65	*Pinus longifolia*	3-Carene	26049
66	*Pinus longifolia*	Lsocineole	10106
67	*Pinus longifolia*	α-Terpinene	7462
68	*Pinus longifolia*	Cymene	7463
69	*Pinus longifolia*	Limonene	22311
70	*Pinus longifolia*	Eucalyptol	2758
71	*Pinus longifolia*	τ-Terpinene	7461
72	*Pinus longifolia*	2-Methyldecane	23415
73	*Pinus longifolia*	Terpinolene	11463
74	*Pinus longifolia*	n-Undecane	14257
75	*Pinus longifolia*	n-Nonanal	31289
76	*Pinus longifolia*	α-Thujone	91456
77	*Pinus longifolia*	Fenchol	15406
78	*Pinus longifolia*	L-trans-Pinocarveol	88302
79	*Pinus longifolia*	Camphor	2537
80	*Pinus longifolia*	Isoborneol	64685
81	*Pinus longifolia*	α-Phellandren-8-ol	519323
83	*Pinus longifolia*	p-Acetyltoluene	8500
84	*Pinus longifolia*	Terpinen-4-ol	11230
85	*Pinus longifolia*	p-Cymen-8-ol	14529
86	*Pinus longifolia*	α-terpineol	442501
87	*Pinus longifolia*	Myrtenal	61130
88	*Pinus longifolia*	n-Decanal	8175
89	*Pinus longifolia*	cis-Carveol	330573
90	*Pinus longifolia*	Resin	11290200
91	*Pinus longifolia*	Cumin aldehyde	326
92	*Pinus longifolia*	n-Nonanoic acid	8158
93	*Pinus longifolia*	Thymol	6989
94	*Pinus longifolia*	Carvacrol	10364
95	*Pinus longifolia*	2,4-Decadienal	5283349
96	*Pinus longifolia*	α-Terpinyl acetate	88693
97	*Pinus longifolia*	α-Longipinene	520957
98	*Pinus longifolia*	n-Decanoic acid	2969
99	*Pinus longifolia*	Longicyclene	564934
100	*Pinus longifolia*	α-Ylangene	101607926
101	*Pinus longifolia*	(+)-Sativen	530427
102	*Pinus longifolia*	β-Caryophyllene	1742210
105	*Pinus longifolia*	n-Undecanoic acid	8180
106	*Pinus longifolia*	Humulene	5281520
107	*Pinus longifolia*	Dodecanoic acid	3893

108	*Pinus longifolia*	(+)-Longicamphenylone	289152
109	*Pinus longifolia*	Globulol	12304985
111	*Pinus longifolia*	Viridiflorol	11996452
113	*Pinus longifolia*	Tridecanoic acid	12530
114	*Pinus longifolia*	Longiverbenone	530428
115	*Pinus longifolia*	α-Santalol	6857681
116	*Pinus longifolia*	Pentadecanal	17697
117	*Sida cordifolia Linn*	Ephedrine	9294
118	*Sida cordifolia Linn*	methyltryptophan methyl ester	14284053
119	*Sida cordifolia Linn*	hypaphorine	442106
120	*Sida cordifolia Linn*	vasicinone	442935
122	*Sida cordifolia Linn*	vasicinol	442934
123	*Sida cordifolia Linn*	choline	305
124	*Sida cordifolia Linn*	Betain	247
125	*Sida cordifolia Linn*	1,2,3,9-tetrahydro-pyrrolo	2394026
126	*Sida cordifolia Linn*	Quinazolin-3-ylamine	46173627
129	*Sida cordifolia Linn*	5,7-dihydroxy-3- isoprenyl flavone	134612824
130	*Sida cordifolia Linn*	5-hydroxy-3-isoprenyl flavone	132278742
131	*Sida cordifolia Linn*	Asparagine	6267
132	*Sida cordifolia Linn*	Rutin	5280805
134	*Sida cordifolia Linn*	Linoleic acid	5280450
135	*Sida cordifolia Linn*	Malvalic acid	10416
136	*Sida cordifolia Linn*	Sterculic acid	12921
137	*Sida cordifolia Linn*	Coronaric acid	6246154
138	*Sida cordifolia Linn*	Mucin	124890
139	*Sida cordifolia Linn*	Pseudoephedrine	7028
140	*Sida cordifolia Linn*	Ecdysterone	5459840
142	*Withania Somnifera*	Somniferine	14106343
143	*Withania Somnifera*	Pseudo-tropine	99088

145	*Withania Somnifera*	Cuscohygrine	1201543
146	*Withania Somnifera*	Isopelletierine	92987
147	*Withania Somnifera*	Anaferine	443143
148	*Withania Somnifera*	Withaferin A	265237
149	*Withania Somnifera*	Withanone	21679027
150	*Withania Somnifera*	Hydroxyproline	5810
151	*Withania Somnifera*	Glutamic acid	33032
152	*Withania Somnifera*	Cystine	595
153	*Withania Somnifera*	Cysteine	5862
157	*Acacia nilotica*	Resin	1183
158	*Acacia nilotica*	Gallic acid	370
159	*Acacia nilotica*	Caffeic acid	689043
160	*Acacia nilotica*	Ellagic acid	5281855
163	*Acacia nilotica*	Hexacosanol	68171
164	*Acacia nilotica*	Triacontanol	68972
166	*Acacia nilotica*	Leucocyanidin	71629
169	*Acacia nilotica*	Protocatechuic acid	72
170	*Acacia nilotica*	Pyrocatechol	289
171	*Acacia nilotica*	Catechin	9064
172	*Acacia nilotica*	Amyrin	73145
175	*Acacia nilotica*	Octaconsanol	ChemSpider ID:61689
176	*Andrographis paniculata*	Andrographolide	5318517
177	*Andrographis paniculata*	14-deoxyandrographolide	11624161
178	*Andrographis paniculata*	neoandrographolide	9848024
179	*Andrographis paniculata*	14-deoxy-11,12-didehydroandrographolide	5708351
180	*Andrographis paniculata*	14-deoxy-14,15-didehydroandrographolide	6473762
181	*Andrographis paniculata*	Andrograpanin	11666871
182	*Andrographis paniculata*	isoandrographolide	343585
183	*Andrographis paniculata*	14-acetylandrographolide	71589914

187	*Andrographis paniculata*	5,2'-dihydroxy-7,8-dimethoxyflavon	5491798
188	*Andrographis paniculata*	Cinnamic acid	444539
189	*Andrographis paniculata*	4-hydroxy-2-methoxycinnamaldehyde	5374604
190	*Andrographis paniculata*	Oleanolic acid	10494
191	*Andrographis paniculata*	β-daucosterol	296119
193	*Andrographis paniculata*	Kalmeghin	ChemSpider ID16735664
194	*Cannabis sativa*	Cannabidiol	644019
195	*Cannabis sativa*	Cannabichromene	30219
196	*Cannabis sativa*	Cannabigerol	5315659
197	*Cannabis sativa*	delta-9-tetrahydrocannabinol	16078
199	*Cannabis sativa*	Friedelan-3-one	91472
200	*Cannabis sativa*	Epifriedelanol	119242
202	*Cannabis sativa*	Ergost-5-en-3-ol	173183
203	*Cannabis sativa*	Palmitic acid	8181
204	*Cannabis sativa*	Pentadecanoic acid	13849
205	*Cannabis sativa*	10E-hexadecenoic acid	ChemSpider ID4471837
207	*Cannabis sativa*	β-sitosterol-β-D-glucoside	5742590
208	*Cannabis sativa*	*p*-coumaroyltyramine	5372945
209	*Cannabis sativa*	Caryophyllene	5281515
210	*Cannabis sativa*	pinene	14896
211	*Cannabis sativa*	Myrcene	31253
212	*Cannabis sativa*	Linalol	6549
215	*Cannabis sativa*	olivetol	10377
216	*Cannabis sativa*	Tetrahydrocannabivarin	93147
217	*Cannabis sativa*	Tetrahydrocannabinolic acid	98523
218	*Cannabis sativa*	Cannabicyclol	30607
219	*Cannabis sativa*	Cannabigerolic acid	6449999
221	*Cannabis sativa*	Cannabinodiol	11551346
222	*Carica Papaya*	Citric acid	311

223	*Carica Papaya*	Malic acid	525
225	*Carica Papaya*	Myristic acid	11005
226	*Carica Papaya*	N-butyric acids	ChemSpider ID259
227	*Carica Papaya*	n-hexanoic	3095157
228	*Carica Papaya*	n-octanoic acids	8091
229	*Carica Papaya*	Oleic acid	445639
230	*Carica Papaya*	(+)-Carpaine	ChemSpider ID390994
231	*Carica Papaya*	Benzylisothiocynate	ChemSpider ID8436906
232	*Carica Papaya*	Benzylglucosinolat	ChemSpider ID7827528
233	*Carica Papaya*	Glucotropacolin	ChemSpider ID32821140
234	*Carica Papaya*	Benzylthiourea	737375
236	*Carica Papaya*	Myrosin	ChemSpider ID:4534
238	*Carica Papaya*	Papain	3705436
239	*Carica Papaya*	Glutamine	5961
240	*Carica Papaya*	Chymopapains A	9001
242	*Citrus lemon*	Neral	643779
245	*Citrus lemon*	*cis*-α-bergamotene	6429303
246	*Citrus lemon*	Geraniol	637566
247	*Citrus lemon*	*trans*-carveol	94221
251	*Citrus lemon*	p-mentha-2,8-dien-1-ol	155626
252	*Citrus lemon*	Estragole	8815
253	*Citrus lemon*	α-fenchene	28930
254	*Citrus lemon*	Perillol	10819
255	*Citrus lemon*	β-curcumene	442360
256	*Citrus lemon*	*trans*-d-limonene oxide	449290
257	*Citrus lemon*	1-naphthalenamine	8640
260	*Citrus lemon*	β-santalene	94164
261	*Citrus lemon*	β-sesquiphellandrene	12315492
264	*Citrus lemon*	3-terpinolenone	381152

265	*Citrus lemon*	Zizaene	527247
266	*Citrus lemon*	Germacrene -D	5317570
267	*Citrus lemon*	Bisabolol	1549992
269	*Citrus lemon*	Citronellal	7794
271	*Citrus lemon*	1-undecanol	8184
		Citric acid	
272	*Citrus lemon*	sabinol	94147
274	*Camellia sinensis*	Caffeine	2519
275	*Camellia sinensis*	Theobromine	5429
277	*Camellia sinensis*	(-)-epiafzelechin-3-O-gallate	467295
278	*Camellia sinensis*	(+)-catechin-3-O-gallate	5276454
279	*Camellia sinensis*	(+)-Afzelechin	442154
280	*Camellia sinensis*	(-)-epicatechin-3-O-p-hydroxybenzoate	10764037
283	*Camellia sinensis*	Myricetin	5281672
284	*Camellia sinensis*	Oleanane	9548717
285	*Arachis hypogaea*	Arachidic acid	10467
286	*Arachis hypogaea*	Aspartic acid	5960
287	*Arachis hypogaea*	Behenic acid	8215
289	*Arachis hypogaea*	Gadoleic acid	5282767
293	*Arachis hypogaea*	Gentisic acid	3469
295	*Arachis hypogaea*	Palmitoleic	445638
296	*Arachis hypogaea*	Ascorbic acid	54670067
297	*Arachis hypogaea*	Caprylic	454067
298	*Arachis hypogaea*	Lecithin	26198
302	*Arachis hypogaea*	Alanine	5950
303	*Arachis hypogaea*	Arginine	6322
305	*Arachis hypogaea*	Phenylalanine	6140
306	*Arachis hypogaea*	Glycine	750
309	*Arachis hypogaea*	1-octen-3-ol	18827
310	*Arachis hypogaea*	Heneicosane	12403

312	*Arachis hypogaea*	4-vinylguaiacol	332
313	*Arachis hypogaea*	Phytol	5280435
314	*Centella asiatica*	Asiatic acid	119034
315	*Centella asiatica*	Asiaticoside A	45356919
316	*Centella asiatica*	Madasiatic acid	23132225
322	*Centella asiatica*	Linolenic acid	5280934
325	*Centella asiatica*	Stearic acids	18962935
326	*Centella asiatica*	Valerin	16760703
330	*Elettaria cardamomum*	Sabinene	18818
332	*Elettaria cardamomum*	6-methyl-5-hepten-2-one	9862
334	*Elettaria cardamomum*	α-phellandrene	443160
339	*Elettaria cardamomum*	*cis*-sabinene hydrate	101629835
340	*Elettaria cardamomum*	*cis*-linalool oxide (furanoid)	22310
342	*Elettaria cardamomum*	2-hexyl furan	566912
344	*Elettaria cardamomum*	*cis*-*p*-menth-2-en-1-ol	122484
346	*Elettaria cardamomum*	Sabina ketone	92784
347	*Elettaria cardamomum*	δ-terpineol	17100
351	*Elettaria cardamomum*	*trans*-piperitol	85568
352	*Elettaria cardamomum*	Carvone	7439
355	*Elettaria cardamomum*	Linalyl acetate	8294
356	*Elettaria cardamomum*	2-phenyl-2-butenal	6429333
358	*Elettaria cardamomum*	*cis*-2.3-pinanediol	62044
359	*Elettaria cardamomum*	Methyl geranate	5365910
360	*Elettaria cardamomum*	Myrtenyl acetate	61262

Table S3 Mapped gene targets with a significant role in MTB aetiology

Functions	Gene ID	PDB Identifier
Virulence factor	Rv3602c	1MOP
	Rv3377c	6VPT
	Rv3378c	3WQK
	HTRA2	1Y8T
	Rv2379c	2KHR
Gene for Cell wall Biosynthesis	Rv1086	2VFW
	Rv2361c	2VG2
	UDP- glf	1V0J
	fadD29	7AHB
	mmaA2	1TPY
Microbial enzymes	Rv2498c	1U5V
	PanK	2GET
	PyrG	4ZDI
	ThyA	3QJ7
	Thyx	2AF6

Table S4 Primers for 1TbAd

No	Primer name	5' Sequence 3'	Nucleotide
**1)**	1TbAd_FP	**CTGCTCAGCTCAGGAAAAAC**	20 Nucleotide
**2)**	1TbAd _RP	**TGATCGTACAAGATCCTCCG**	20 Nucleotide

Table S5 Mutation in the familiar residue of the 1-TbAD

Wild type	Residue Position	Mutant type	DynaMut predicted ΔΔG	Docking score
GLY	35	CYS	0.817 kcal/mol (Stabilizing)	-9.6
GLY	35	GLU	-0.56 kcal/mol (Destabilizing)	-9.3
GLY	35	HIS	-1.06 kcal/mol (Destabilizing)	-9.8
GLY	35	ILE	1.07 kcal/mol (Stabilizing)	-9.7

GLY	35	LEU	1.12 kcal/mol (Stabilizing)	-9.4
TYR	90	GLU	-0.73 kcal/mol (Destabilizing)	-9.3
TYR	90	HIS	-1.2 kcal/mol (Destabilizing)	-9.3
TYR	90	THR	-0.58 kcal/mol (Destabilizing)	-9.2
TYR	90	TRP	-1.51 kcal/mol (Destabilizing)	-9.4
TYR	90	VAL	1.36 kcal/mol (Stabilizing)	-9.3
THR	36	ASP	0.816 Kcal/mol (Stabilizing)	-10.2
THR	36	LYS	0.566 kcal/mol (Stabilizing)	-9.4
THR	36	SER	-0.452 kcal/mol (Destabilizing)	-10.3
THR	36	TRP	1.342 kcal/mol (Stabilizing)	-9.5
THR	36	VAL	-0.09 kcal/mol (Destabilizing)	-10.1

Table S6 Mutation in the familiar residue of the rpoB

**Wild type**	**Residue Position**	**Mutant type**	**DynaMut predicted ΔΔG**	**Docking score**
ARG	222	CYS	0.49	-8.9
ARG	222	GLU	-0.23	-7
ARG	222	HIS	-1.29	-7.8
ARG	222	ILE	-0.94	-9
ARG	222	LEU	0.78	-8.5

Table S7 Prediction of the active binding pockets for 1-TbAd-3WQK

No	Receptor	PDB ID	Centre			Size			Residues	Docking score
			X	Y	Z	X	Y	Z		
1	1TbAd	3WQK	-9.26	-13.1	10.38	24	19	21	TYR90,ILE233,GLY35	-11.8
2	1TbAd	3WQK	-28.1	-2.54	-1.80	32	32	32	TYR90,THR36,ASP34,SER219	-10.5
3	1TbAd	3WQK	-29.6	-28.8	12.39	26	40	40	TYR90,GLY35,TYR51,ILE78,TYR233	-10
4	1TbAd	3WQK	-16.6	-19.8	20.33	20	26	21	SER80,THR36,GLY35,ARG22	-9.4
5	1TbAd	3WQK	-26.4	-6.25	26.26	30	26	24	PHE141,TYR124,PHE120,PHE172,SER168,GLY122,TYR124	-9.2
6	rpoB	4KBJ	40.70	62.2	109.4	27	33	35	ARG222,LYS223	-9.2
7	rpoB	4KBJ	66.26	61.08	70.54	27	27	35	GLY203	-8.7
8	rpoB	4KBJ	62.33	76.82	111.0	27	27	27	ARG222	-8
9	rpoB	4KBJ	76.50	73.46	85.54	27	27	27	ASP73,GLU377,ARG71	-7.8
10	rpoB	4KBJ	78.01	42.73	79.21	27	27	27	THR326	-7

Additional file 1 Figure

(a)

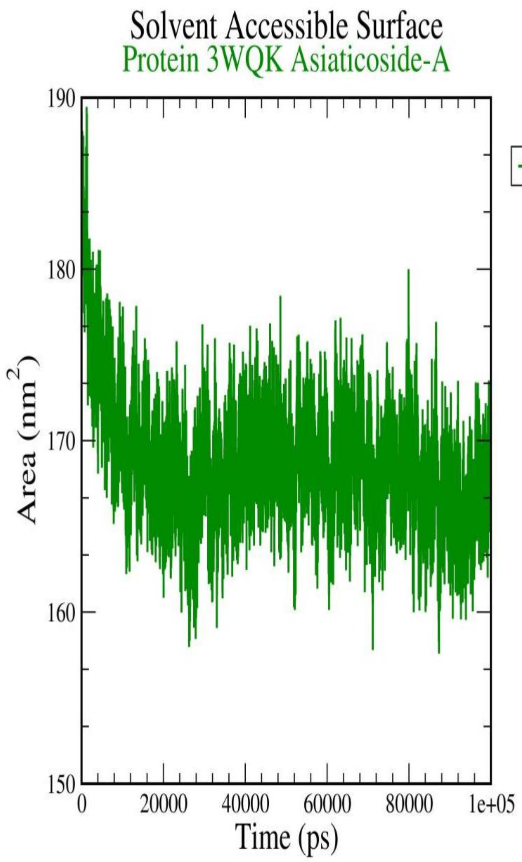

(b)

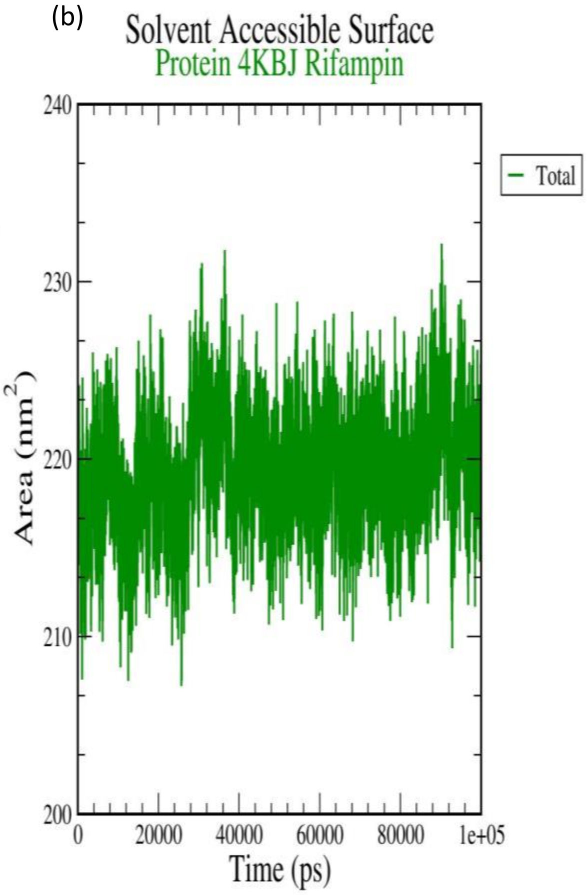

Figure S1 A plot SASA Value (A) 3WQK_ASA (B) rpoB_Rifampin

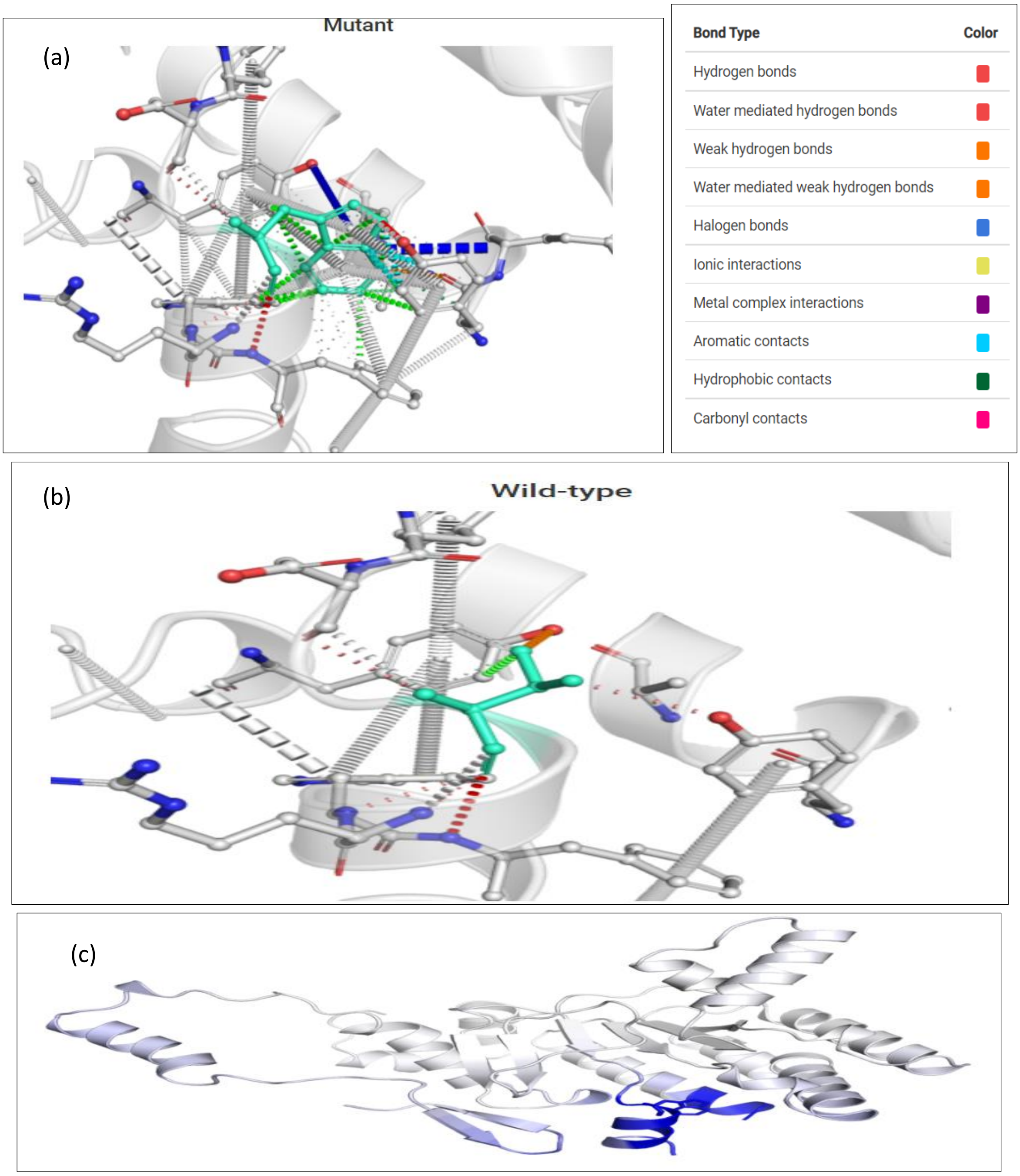

Figure S2 (a) Wild type (b) Color codes are displayed on the right panel, and mutant residue is colored in the light-green and depicted in the stick. (c) The vibrational entropy change that occurs during mutation causes amino acids to change color. The rigidification of the structure is represented by the color blue.
